# Patients’ views of adverse events in primary and ambulatory care: a systematic review to assess methods and the content of what patients consider to be adverse events

**DOI:** 10.1186/s12875-016-0408-0

**Published:** 2016-01-27

**Authors:** Sarah Lang, Marcial Velasco Garrido, Christoph Heintze

**Affiliations:** Institute for General Practice und Family Medicine, Charité, Universitätsmedizin Berlin, Charitéplatz 1, D- 10117 Berlin, Germany; Institute for Occupational and Maritime Medicine, University Hamburg-Eppendorf, Berlin, Germany

**Keywords:** Medical errors, Adverse events, Patient safety, Primary health care, Ambulatory care, Patients perspective, Public opinion, Patients views

## Abstract

**Background:**

Patient safety gained widespread public attention in the last 20 years. However, most patient safety research relied upon professionals’ exceptions and was realised especially in the hospital sector. Gradually patients’ attention has been focused on safety campaigns in inpatient care. We aimed to better assess patients’ perceptions in primary and ambulatory care.

**Methods:**

A systematic review was conducted by use of database searches with additional reference and hand searching. The search strategy implied MeSH-terms relating to adverse events, incident reporting and outpatient care. Relevant articles were selected by applying defined eligibility criteria. Studies exclusively based on hospital data as well as the professionals’ point of view were excluded.

**Results:**

We included 19 studies. Patients were able to identify events that were traditionally recognised by the medical community as technical medical aspects (e.g. errors in diagnosis). An important field of patient participation in prevention of adverse events was proposed in the medication process. Most reported events however could be described as service quality incidents. Communication problems were shown to have implications on the occurrence of technical medical aspects and patients’ satisfaction of their care. Further, unsatisfied patients were more likely to recognize adverse events.

**Conclusion:**

Patients’ perception of patient safety in primary and ambulatorycare broadened the previous focus on technical medical aspects. Especially communication factors played an important role in the occurrence and consequence of adverse events and patients’ satisfaction. Future research should concentrate on developing possible ways to integrate patients’ views and participation in ensuring safety in outpatient care.

## Background

There has been growing interest in patient safety within the last 20 years. Especially, since the release of the Institute of Medicine (IOM) report “To Err Is Human” in 1999 the issue gained widespread public attention and the number of patient safety publications has been continuously increasing [1]. Safety campaigns and strategies developed in the years following the IOM report focused mainly on the perspective of health care professionals in the inpatient sector [2]. However, health care is still mostly delivered in the ambulatory sector and primary care, which is commonly the basis of patients’ health care [3–5]. Some characteristics of outpatient care - such as short consultation times, frequency of chronic diseases, lack of communication with the hospital sector and between ambulatory professionals - might lead to a specific risk environment for adverse events [6]. Studies addressing patient safety in outpatient settings have used a broad variety of definitions for adverse event, measurement methods and taxonomies that hinder comparison, considering the lack of a unique international terminology and classification system [7].

Recent projects to understand the nature of medical errors in primary care have been based on physicians’ perspective, e.g. the “Primary Care International Study on Medical Errors” (PCISME) [8] and the “Applied Strategies for Improving Patient Safety” (ASIPS)-Project [9]. The Linnaeus-Euro-PC collaboration which represents eight research institutions and patient safety organizations from six European countries is doing essential work on improving patient safety in primary European care. Among its objectives are the development of a taxonomy of errors in primary care, the implementation of a standardized reporting system, and methods to measure patients’ perspective of safety issues and patients’ involvement in safety initiatives. In the last decade patient safety research has discussed gradually the importance of patients’ views and their role in ensuring their own safety [10–12].

In this systematic review, we aimed to produce a comprehensive summary of the published literature assessing patients’ views on adverse events in primary and ambulatory care. As secondary objectives, we intended to (A) better characterize the methods used by the researchers; and (B) report the solutions proposed to increase the participation of patient in their own care.

## Methods

### Data Sources

This review was designed and reported according to the PRISMA statement “TRANSPARENT REPORTING of SYSTEMATIC REVIEWS and META-ANALYSIS –PRISMA [13]. We performed a systematic literature search on the databases: MEDLINE, OvidSP, CINAHL, Cochrane Library, PsycInfo and ScienceDirect, from the earliest available date to August, 2012. Publications in English, French or German-language were included. We used Medical Subject Headings (MeSH) terms and free text terms related to patient safety themes, as illustrated in Table 1.

**Table 1:** Search strategy in MEDLINE

Search	Search strategy
#1	ambulatory care OR primary health care OR outpatients OR family practice OR ambulatory specialty
#2	medical error OR harm OR adverse events OR preventable adverse events OR iatrogenic disease OR medical injury OR malpractice OR near miss OR medication error OR adverse drug event OR adverse drug reaction OR patient safety OR safety incident OR disclosure
#3	patients perspective OR patient report OR patient opinion OR public opinion OR public view OR patient experiences
#4	#1 AND #2 AND #3

Additional articles were found through reference lists checking of specialized journals (e.g. *BMJ Quality and Safety*, *Family Practice*, *Archives of Family Medicine*), internet hand searches, and by exploring internet sites of patient safety organizations (e.g. Linneaus-Euro-PC collaboration, the World Health Organization, the National Patient Safety Agency and the Joint Commission on Accreditation of Healthcare Organizations) for ongoing projects and “grey literature”.

### Study selection

First, all screened titles and abstracts were reviewed by one of the authors (SL) and were considered eligible if they provided original data on patients’ views of adverse events in primary and ambulatory care settings, with or without comparison to health care professionals’ perspective. The selection process was checked by a second author (CH). Qualitative as well as quantitative research approaches were accepted. Articles were excluded if [1] they investigated only health care professionals’ perspective; [2] addressed the hospital setting; [3] reported only patients’ satisfaction, shared decision-making or other aspects of quality of care than adverse events. In a second step, eligibility was confirmed with full-text review. We did not include reviews, essays, and editorials.

### Data extraction

Included articles were abstracted for publication metadata, country, type of healthcare setting, socio-demographic characteristics of sample, and results concerning patients’ perspective on adverse events. Particularly, methods used to interrogate participants’ opinions were analysed using an adapted version of Schwartz’s interview structure (demonstrated in Table 2), in order to specify to which extent patients were free to express their opinion (through structured or unstructured questions and open- or close-ended answers) [13]. As we included both qualitative and quantitative studies, we could not apply a single standard instrument suitable for a global quality assessment [5]. We thus decided to assess the quality of the included studies by checking whether authors presented full insight on sample characteristics, data extraction (e.g. interview or questionnaire type), analysis methods, and discussed limitations.

**Table 2:** Interview structures according to Schwartz et al. [14] with common examples^a^

Questions	Response options^b^		
	Close-ended	Partly open-ended	Open-ended
Structured	type 1 - written questionnaire - face-to-face interview - telephone interview	type 2 (as type 1)	type 3 (as type 1)
Semi-structured	type 4 - telephone interview	type 5 - problem-centered interview	type 6 - expert interview - focused interview
Unstructured	type 7	type 8	type 9 - narrative interview - in-depth interview

^a^This classification was used for the initial assessment of included studies

^b^ Response options according to Schwartz et al. [14]: close-ended implies prior determination of possible answers; partly open-ended allows addition of responses other than the presetted ones; open-ended response option leaves the respondant to answer freely

## Results

### Overview

**Fig. 1:**
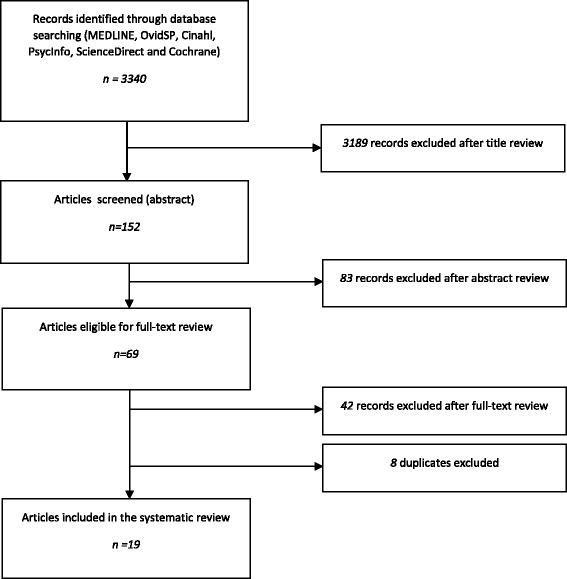
Selection of articles

The literature research returned 3340 candidate articles of which 68 were selected for full-text-review. Twenty-six publications met all eligibility criteria. After exclusion of duplicates, 19 original research studies remained and were included in this systematic review. These were 12 quantitative [14–25] and 7 qualitative [26–32] studies. Figure 1 shows the selection process.

Eightteen out of 19 studies were published after 2004. Most of the research has been undertaken in the US (68 %) and only one publication compared results from different countries [19]. Observed variety of outpatient care settings depended on country-specific health care systems. Demographic characteristics of study participants were given in 13 publications and consisted of information about the age, cultural background, personal knowledge, income and insurance situation [14, 16–18, 22–24, 26–31]. The number of participating patients and demographic information was too heterogeneous to draw significant conclusions regarding gender, age and sociocultural differences.

### Research design and analytical approach

Table 3 shows characteristics and methods of data collection among included studies. In 16 of the papers [except for [14, 15, 25]], primary data was collected using personal or telephone interviews, focus groups or written surveys. The structure of patient interrogation differed depending on qualitative and quantitative research concepts. Qualitative studies used structured, partly structured or unstructured interviews of individuals next to focus groups emphasizing group dynamic process. Response options were partly open-ended answers or additional free text lines. In comparison, all quantitative research papers interrogated patients with structured question designs and provided partly open- or close-ended answering possibilities. In two articles further interviews or additional free-text options were used to specify patients’ responses [18, 22]. Three publications reported analysis of malpractice claims as secondary data [14, 15, 25]. These studies can be considered to provide an indirect summary of patient’s opinion, since claim files include complaint statements as well as multiple third-party documents (e.g. reports of investigations, legal records) which provide insights in the patient view. Two publications combined primary and secondary data analysis using incident reporting and chart review [16, 17]. Five of the 16 primary data studies compared patients’ views with the professional opinions of caregivers [16, 17, 19, 21, 26, 27] and with patient records [16, 17].

**Table 3:** Characteristics and methods of data collection

Study	Country^a^	Setting	N	Data collection	Focus^b^	Definition of adverse events	Questioning design	Answering design according to Schwartz
Brown, 2006 [29]	USA	Primary care (not specified)	22	Individual interview	G	Other	Semi-structured	Open-ended
Buetow, 2009 [26]	NZ	Primary care (not specified)	64	Focus group	G	Reason [23]	Unstructured	Open-ended
Buetow, 2010 [27]	NZ	Primary care (not specified)	64	Focus group	G	Reason [23]	Semi-structured	Partly open-ended
Dowell, 2005 [28]	USA	Primary care (not specified)	21	Focus group	P	NA	Structured	Partly open-ended
Elder, 2005 [30]	USA	University-affiliated, community based family practices	24	Individual interview	P	NA	Structured	Partly open-ended
Gandhi, 2006 [15]	USA	Physicians office, ambulatory surgery, radiology sites, emergency department	NA	Malpractice claims	P	IOM [25]	NA	NA
Gaal, 2011 [49]	NL	Family physicians	250	Malpractice claims	P	NA	NA	NA
Kistler, 2010 [19]	USA	Primary care (medical practices)	1,697	Survey (tel.)	P	Other	Structured	Partly open-ended
Kuzel, 2004 [31]	USA	General internists, pediatricians, family physicians	38	Individual interview	P	Other	Structured	Partly open-ended
Phillips, 2004 [16]	USA	Family physicians, general internists, general pediatricians	49,345	Malpractice claims	P	NA	NA	NA
Phillips, 2006 [22]	USA	General physicians in private practices or residency clinics	NA	Survey (written, internet or tel.)	P	IOM [25]	Structured (for doctors, NA for patients)	Partly open-ended (for doctors, NA for patients)
Schoen, 2004 [20]	AUS, CAN, NZ, UK, USA	Primary care (not specified)	7,200	Survey (tel.)	P	NA	Structured	Close-ended
Solberg, 2008 [23]	USA	Physician multispecialty group	1,998	Survey (written)	P	IOM [25]	Structured	Partly open-ended
Tam, 2008 [17]	China	Primary care clinics	600	Survey (tel.); Voluntary reports; Chart review	P	Other	Structured	Partly open-ended
Unruh, 2006 [32]	USA	Outpatient cancer care	18	Individual interview	P	Reason [23]	NA	NA
Wasson, 2007 [24]	USA	Clinical practices	44,860	Survey (internet)	P	Other	Structured	Partly open-ended
Weingart, 2007 [21]	USA	Cancer center	193	Individual interview	P	NA	Structured	Partly open-ended
Wetzels, 2008 [18]	NL	Family physicians	50	Survey (written); GP & pharmacist reports; Medical and autopsy records	P	WHO [24]	NA	Close-ended
Witman, 1996 [25]	USA	General internal medicine outpatient clinic of a university medical center	149	Survey(written)	G	NA	Structured	Partly open-ended

*NA* not available

^*a*^ *NZ* New Zealand, *AUS* Australia, *CAN* Canada, *UK* United Kingdom, *NL* Netherlands

^b^ *G* general attitudes, *P* personal experience

Concerning terminology of adverse events, the term “medical errors” was used next to “mistakes”, “adverse events”, “unsafe care”, “all events with resulting harm” or “preventable problems”. Kistler et al. [18, 19] reported that patients preferred the term “mistakes” to “medical errors”. Ten studies (56 %) applied international definitions [e.g. Reason, WHO [14, 16, 21, 22, 26, 27, 32] or individual concepts [23, 29, 31]].

Fifteen publications focused on patients’ experiences with adverse events in primary care whereas the remaining four studies [24, 26, 27, 29] explored general attitudes on this subject. In all but two of the articles [26, 27] patients were asked to report adverse events caused by doctors. In four articles, specific kinds of adverse events were analysed: errors in diagnosis and treatment [18, 22], dispensing and intervention [22], diagnosis [19] and medication [16].

### Quality assessment of included studies

The quality of the studies was assessed by checking whether critical features were found in the articles. Overall, reporting quality was good in the majority of publications. Sixteen publications (88 %) reported all of the following elements: research questions and study objectives, study design, research team expertise and task and, analysis methods. These studies provided also information on the development process of the questionnaire used, including piloting, as well as on the content of the questionnaire. In these studies, results were presented according to study protocol. Two studies [17, 32] did not fully display the questionnaire, and one study [32] reported results for only 5 of 18 patients. Fourteen studies discussed the limitations of their work. These included issues on study design [16–18, 20, 22, 24–27], and on reproducibility [14, 15] as well as limited transferability of the results due to the specificity of the study setting [14, 18, 20, 22, 24]. Authors discussed the potential limitations through selection [14, 18, 20, 23, 24, 26–31], interpretation [21, 25], and educational [16, 20, 23, 27, 30] bias.

### Patients’ views of adverse events in primary care

Twelve of 16 studies contained information about types of adverse events caused by doctors in primary care [15–23, 26, 28, 30, 31]. According to patients’ perspective, the research included in the review allows to identify two main groups of adverse events:

First, so named technical medical aspects consisted of errors in diagnosis, treatment, intervention and medication process which are mostly identified by health care professionals. Kuzel et al. [31] recognized that these types of events had been the focus of patient safety campaigns so far. Patients were considered to be able to identify this category to a certain extent.

However, patients mostly identified problems that could be classified as service and quality related problems of primary and ambulatory health care: deficits in doctor-patient-relationship (lack of respect, time pressure, rudeness, break of confidence), coordination, access (long waiting time, no appointments available) and communication (between doctor and patient, among health care professionals). All qualitative studies illustrated in detail that this kind of deficits predominated patients’ understanding of threats to their safety. In six quantitative publications aspects of service and quality problems were addressed [17, 18, 20–23], among three of them by using additional questions [18, 22] or additional free-text-answer or comments possibilities [20].

Furthermore, authors recognised service quality problems as causes or contributing factors to technical medical errors [14, 15, 29], e.g. prescriber-patient miscommunication leads to ambulatory adverse drug events [29].

Concerning the consequences of adverse events, qualitative and quantitative studies discussed different aspects. Qualitative studies distinguished psychological and physiological harm of adverse events [30–32]. The first category contained patients’ emotional responses (e.g. anger, frustration, mistrust) and was more frequent than physiological consequences. Patients’ behaviour was characterised by passive (avoidance or accommodation) and active actions (anticipation and advocacy). Authors noticed a link between patients’ behaviour and the nature of harm: mistrust was associated more likely with avoidance strategies. In contrast, quantitative publications used scales to measure the level of harm induced by adverse events and rated most events as significant or severe [14–16, 18]. Patients were also asked about physician behaviours after adverse events [24]. They reported their wish for disclosure of even minor errors and referral to another doctor following severe events. Authors showed that patients’ intention of litigation was lower when physicians were willing to disclose or openly disclosed errors [24].

### Importance of patients’ perspective for patient safety

Seven publications acknowledged the importance of patients’ perspective for measuring adverse events [16, 17, 20–23, 28]. The main advantage was seen in detecting service and quality problems which could themselves lead or contribute to technical medical aspects. However, uncertainty was expressed concerning the adequate measurement tool to integrate this perspective in daily routine of outpatient care. Three authors proposed for efficient measurement of adverse events the combination of different tools like professional opinion, chart review, medical records and patients’ opinion [16, 17, 21].

In one of the papers, a link was observed between service and quality incidents to decreased satisfaction of patients which further could lead to increased appearance and perception of adverse events [22]. Though, the included literature emphasised not to mix patient surveys about satisfaction and quality of health care with those about surveillance of adverse events.

## Discussion

Patients’ perspective of safety incidents showed both overlaps and additional aspects from outpatient care professionals’ opinions. The integration of patients’ perspective can lead to better understanding of patient safety in primary and ambulatory care. Patients’ statements brought insight on the nature, causes, and consequences of adverse events and could be considered as an efficient measurement of adverse events, as already proved for the inpatient sector [33]. Especially in ambulatory care, where patients represent the most continuous member in an often fragmented health care process, patients’ point of view can contribute efficiently in ensuring their own safety.

Patients’ reports revealed some technical medical aspects that were mostly named by professionals: delayed or wrong diagnosis, medication dispensing errors, improper interventions. The extent to which patients are able and willing to assess these factors meaningfully still needs to be explored.

Patients were mostly concerned about service and quality problems within their health care, which they see as safety threats [47]. Interestingly, doctors tended to explain the occurrence of technical medical aspects by deficits in organisation or administration and communication problems. A positive development within the last years can be seen in the increasing professional perception of service and quality aspects as important types of adverse events: the PCISME study [8] named process errors as most frequent events in general medicine and the ASIPS project [9] identified communication problems in 71 % of reported adverse events.

In our review, patients highlighted communication problems. The role of communication in the occurrence of adverse events is discussed increasingly in patient safety research. In outpatient care, communication structure contains different levels beyond doctor-patient relationship: administration and organisation in the office, contact with other private and institutional health care providers. Our review showed that deficits in communication from the doctors’ side could be reported as the only safety issue (e.g. overuse of medical vocabulary), but could also worsen existing problems (e.g. lack of explanation when test results are late). On the other side, patients with communication incapacities were judged to be at higher risk of adverse events as they seemed to suffer more often from depression and other comorbidities, which themselves represent a risk for the occurrence of adverse events [34]. Comparing with existing literature we conclude that an efficient communication could have positive consequences in different ways: (I) direct prevention of adverse events [34–36]; (II) reducing psychological distress for the patients [30, 37]; (III) increased patient satisfaction, and therefore (IV) reduced susceptibility to adverse events [22], misinterpretation of normal challenges in diagnosis or treatment [18] and thus decreased number of malpractice claims [24, 37]. The efforts to realize patient involvement struggle with still widespread hierarchical unidirectional concepts of delivering care. In addition, they have to struggle with doctor’s fear of being used. Therefore, there is a need for social acceptance and trust in order to increase doctors’ willingness to improve communication, particularly when an adverse event occurs. Communication guidelines have not only been developed for medical caregivers but increasingly for patients, Propositions consisted in ameliorating information of patients about their health status and treatment plan, and on the other side in reporting adverse events and reactions related to the care. However, there is no existing evidence of adequacy of these guidelines for the specific sector of outpatient care.

Emphasizing patients’ perspective in detecting adverse events in outpatient care led to the question of whether and how patients could take part beyond reporting. Patient involvement in error prevention has been increasingly discussed in the literature and different patient safety campaigns have promoted possible actions, like patients ensuring hand disinfection within the medical team. Ambulatory care is a suitable setting for patient involvement in ensuring its own safety [34]. For successful patient action patients’ willingness and capacity, social determinants as well as appreciation by the medical community need to be considered [11, 12, 38, 39, 46, 48].

Only a few authors of the included studies proposed comprehensive involvement. Two studies emphasized patients’ role in ensuring medication safety in primary care [16, 32]. Suggested actions consisted of monitoring side effects, ensuring the correct medication dose and uncovering dispensing errors. Unfortunately, these propositions were not discussed in detail. Many strategies have been developed so far to ensure medication safety in primary care that are mainly addressed to doctors (e.g. using computerized drug interaction alerts, actively communicating with pharmacists, participating in error reporting systems). It is not surprising that patients were considered to be unable to distinguish between medication errors, adverse drug events and undesired effects of a correctly prescribed drug [40, 41]. However, literature appreciated increasingly the benefit of integrating patient adapted methods to prevent adverse events. The best way for detection and prevention of adverse drug events seemed to be a combination of different methods regarding doctors and pharmacists, and improved doctor-patient relationship as condition for all successful patients’ activities.

There is still no agreement about the best way to integrate patients’ perspective in daily outpatient care. We suggest that patients’ reporting use different channels, like paper-based questionaries’ provided in the doctors’ office, or web-based error reporting tools adapted to patients. In the German ambulatory sector, the research group around Barbara Hoffmann has developed a reporting tool “every error counts” for general practitioners [42]. However, little information exists on how patients can use this method. In The Netherlands there are internet-based reporting tools available for patients to address problems related to prescription and medication. However, there is a lack of information about the applicability and actual advantages in daily health care. Two of the included publications promoted web-based reporting tools for patients, but noted the lack of knowledge on how to integrate it in primary and ambulatory care settings [21, 23].

Regarding the design of the included studies, a combination of close-ended answers with free-text lines seems to be the most effective in soliciting patients’ reports, since it allows to check agreement between patients and health care workers on the definition of an adverse event [43]. Hence, in our review, qualitative studies with open-ended answers allowed patients to present their perceptions without being too influenced. However, authors emphasized that in those cases data collection and analysis was very labour-intensive and difficult to integrate in the daily medical routine. On the opposite, authors who used quantitative study designs with close-ended answers intended to confirm rates of existing categories [44]. Therefore, with those quantitative approaches, it was not possible to assess patients’ understanding of adverse events.

Furthermore, there is a need for the use of understandable language in communication with patients. Although there is knowledge that using medical terminology affects patients’ comprehension and behaviour, only two of the included studies demonstrated the influence of specific language used by healthcare workers on patients’ willingness to report [18, 21]. The included studies confirmed previous observations that patients seemed to prefer the term “mistake” to “medical errors” [10].

In addition we underline the difference in asking patients for “experiences” with or “attitudes” towards adverse events. General attitudes are formed by many different factors which haven’t sufficiently been examined so far, like the media, doctors’ information, family values and experiences. Comparisons of patients’ reports in this review were also difficult because of the wide range of demographic characteristics and differences in the social and cultural background. Particularly, higher rates of adverse events are expected with advanced age or chronic diseases. Additionally, differences of reporting between women and men have been demonstrated [35, 45]. None of the analysed studies examined in detail the influence of these factors on patient reports. Concerning the contents, reporting of adverse events by patients should not be mixed with patients’ satisfaction questionnaires, as there is an observed overlap, especially with service quality incidents.

This review has some limitations. The heterogeneity in study design, terminology and measurement tools made comparisons across studies difficult. Besides, heterogeneity did not allow to perform pooled or stratified statistical analysis of results, i.e. regarding the different methodological approaches. We formed substantial clusters of patients’ statements and examined methods of data collection and analysis in a qualitative way.

Most of the included studies have been conducted in US-American outpatient care settings, thus the transferability of results to other health care settings is limited. There is a need to address this topic in the context of European primary health care settings in order to take country specific actions into consideration and realise national and international projects. The Linnaeus-Euro-PC collaboration is amongst others working on developing methods of involving patients in ensuring patient safety (work package nr 8), but we did not found explicit exploring of patients’ concepts of adverse events,

Finally, we included studies addressing malpractice claims. It might be controversial, whether malpractice claims should be counted as patient reported incidents. At least some malpractice claims arise after the patient getting the correct diagnosis or the right therapy from another health care provider, which leads to the perspective of having been the subject of an error. Thus, it could be considered that the error experience is triggered by a health care professional and not the sole experience of a patient. Nevertheless, this kinds of studies provide insight in the experience of patients after knowing the have been subject of an error and may show the discrepancies between what professionals and patients consider to be an adverse event. Thus we considered them relevant for our review.

## Conclusion

Integrating patients’ perspective broadens the existing understanding of adverse events in outpatient care and should therefore be considered as a complimentary measuring tool. Most of the problems identified from the patients perspective were concerns about doctor-patient communication and limitations in coordination or access to health care. The link between potential or real adverse events in ambulant care and concerns or observations of affected patients is still unknown, and should be the object of future research. However, it seems reasonable that flaws of ambulant health care discussed in this paper might foster the development of critical incidents potentially harming for patients.

Our results suggest that patient safety does not only consist of prevention from technical medical errors but also includes the wide range of service and quality problems. Particularly, the patients’ perspective highlights communication issues as a relevant factor for the incidence and severity of adverse events. Patients’ views seem to be best depicted by using a combination of close-ended questions and open-ended narratives. Successful patient involvement – including complex actions, e.g. in the medication process – implies improved physician-patient communication and consideration of patients’ background and wishes. Comparison across countries still keeps limited because of the lack of an international terminology and classification system.

### Ethics

No ethical approval necessary.
